# HPLC-DPPH Screening Method for Evaluation of Antioxidant Compounds Extracted from Semen Oroxyli

**DOI:** 10.3390/molecules19044409

**Published:** 2014-04-10

**Authors:** Renyi Yan, Yangyang Cao, Bin Yang

**Affiliations:** 1 Institute of Chinese Materia Medica, China Academy of Chinese Medical Sciences, Beijing 100700, China; E-Mails: yanry2009@163.com (R.Y.); caoyangyanghebei@163.com (Y.C.); 2 State Key Laboratory of Dao-di Herbs, China Academy of Chinese Medical Sciences, Beijing 100700, China

**Keywords:** *Oroxylum indicum*, flavonoid, HPLC-DPPH, antioxidant, rapid screening

## Abstract

Semen Oroxyli, derived from the seed of *Oroxylum indicum* L., is a commonly used Traditional Chinese Medicine with beneficial effects against several respiratory disorders. Antioxidative flavonoids may be partly responsible for its medicinal functions. The aim of this study was to rapidly determine the antioxidants in Semen Oroxyli based on a HPLC-DPPH method. Four major flavonoids, baicalein-7-*O*-gentiobioside, baicalein-7-*O*-glucoside, baicalein, and baicalin, were identified as the active components against DPPH free radicals, which is in accord with the results of our former traditional activity-guided phytochemical study. The oxidative products of the four antioxidant flavonoids were studied in the DPPH spiking HPLC assay, it was suggested that the three active flavonoid glycosides were converted into 5,6-dihydroxy-7-methoxyflavone, which implied that an additional hydroxyl at C-6 in 5,7-dihydroxyflavones plays an important role in the DPPH assay.

## 1. Introduction

Semen Oroxyli, derived from the seed of *Oroxylum indicum* L., is a commonly used Traditional Chinese Medicine treatment for coughs, chronic pharyngitis, acute bronchitis, upper respiratory tract infections and other respiratory disorders [1,2]. Several compounds have been isolated from *O. indicum*, among of which, flavonoids are the main type [3,4,5,6,7,8,9]. Modern pharmacological studies have showed that polyphenols such as flavonoids possessing antioxidant and anti-inflammatory activities are beneficial to lung inflammation and related diseases [10,11]. Based on these results, the anti-oxidative flavonoids may be partly responsible for the medicinal functions of Semen Oroxyli.

**Figure 1:**
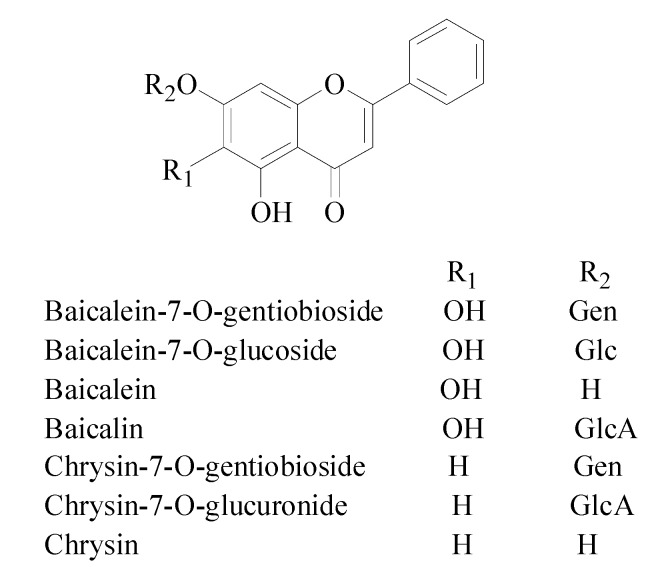
Structures of the seven major flavonoids detected in Semen Oroxyli.

In our laboratory, a traditional activity-guided isolation of antioxidants from Semen Oroxyli has been reported [12]. A successful HPLC method for the quantitative analysis of Semen Oroxyli has been established by us [13]. Studies have shown that seven major flavonoids, baicalein-7-*O*-gentiobioside, baicalein-7-*O*-glucoside, baicalein, baicalin, chrysin-7-*O*-gentiobioside, chrysin-7-*O*-glucuronide, and chrysin (Figure 1) exhibited potent anti-oxidative activities in DPPH or ORAC assays. However, these conventional strategies for identification of antioxidants from complex mixtures are time-consuming, labor intensive, and expensive. In the past few years, online HPLC-DPPH assays have been developed and applied successfully for rapid screening and identification of antioxidants from the crude extracts of herbal medicines [14,15,16]. In the present study, an online post-column HPLC-DPPH assay has been pursued to rapidly screen the antioxidants from Semen Oroxyli. Four major flavonoids, baicalein-7-*O*-gentiobioside, baicalein-7-*O*-glucoside, baicalein, and baicalin, were identified as the active components against DPPH free radicals. In addition, the oxidative products of four antioxidant flavonoids were studied in a DPPH-spiking HPLC assay, and a plausible mechanism was suggested.

## 2. Results and Discussion

### 2.1. Online Post-Column HPLC-DPPH Method

In the online post-column HPLC-DPPH assay, absorbance changes at 515 nm due to the reaction of antioxidants with the DPPH free radical were recorded as negative peaks. Several parameters could affect the sensitivity, such as the concentration of DPPH, oxygen content in the DPPH solution and reaction time [17,18]. Therefore the relevant parameters were optimized to get higher sensitivity. DPPH solution was filtered under vacuum before use to diminish the oxygen content. In Table 1, the influence of DPPH solution concentration on the signal-to-noise ratio (S/N) values for the tested antioxidant baicalein-7-*O*-gentiobioside, was checked with six different DPPH concentrations. Tests were carried out at a flow rate of 0.4 mL/min for the DPPH solution and 1.0 mL/min for the HPLC mobile phase. The best S/N values were obtained when 1 × 10^−5^ M DPPH was used. Ten microliters of the baicalein-7-*O*-gentiobioside were injected into the system using 1 × 10^−5^ M DPPH under different flow rates. The results showed that S/N values increased as flow rate of DPPH solution increased (Table 1). Due to the back pressure soar with increasing flow rate of DPPH solution, a compromising flow rate 0.4 mL/min was then chosen for further experiments.

**Table 1:** The signal-to-noise ratio (S/N) values of baicalein-7-*O*-gentiobioside under different concentrations and flow rates of DPPH.

Concentration of DPPH (mol/L)	S/N	Flow rate of DPPH (mL/min)	S/N
1 × 10^−4^	19.35	0.2	19.84
5 × 10^−5^	18.00	0.3	42.99
1 × 10^−5^	23.67	0.4	53.84
5 × 10^−6^	9.56	0.6	76.36
1 × 10^−6^	ND	0.8	133.43
1 × 10^−7^	ND	1.0	255.27

ND: Not detected.

Based on the set flow rate and concentration of DPPH solution, a method validation was performed on parameters such as linearity, precision, repeatability and stability. The results listed in Table 2.

**Table 2:** Linearity, LOD, LOQ, repeatability, precision, and stability for four antioxidants in online post-column DPPH assay.

	Linearity			LOD (μg)	LOQ (μg)	Repeatability (n = 6)	Precision (n = 6)	Stability (24 h)
	Test range (μg)	Regression Equation	*r*	S/N = 3	S/N = 10	RSD (%)	RSD (%)	RSD (%)
Baicalein-7-*O*-gentiobioside	1.85–18.48	*Y* = 27803.40*X* + 10715.58	0.9991	1.16	1.85	1.58	2.27	4.94
Baicalein-7-*O*-glucoside	1.29–12.90	*Y* = 54206.71*X* − 21149.04	0.9990	0.97	1.29	2.29	3.73	2.58
Baicalin	1.24–12.42	*Y* = 54134.19*X* − 26188.18	0.9992	0.87	1.24	4.24	0.90	2.17
Baicalein	2.50–7.50	*Y* = 49422.32*X* − 39602.40	0.9992	0.06	0.57	4.50	2.17	2.43

**Figure 2:**
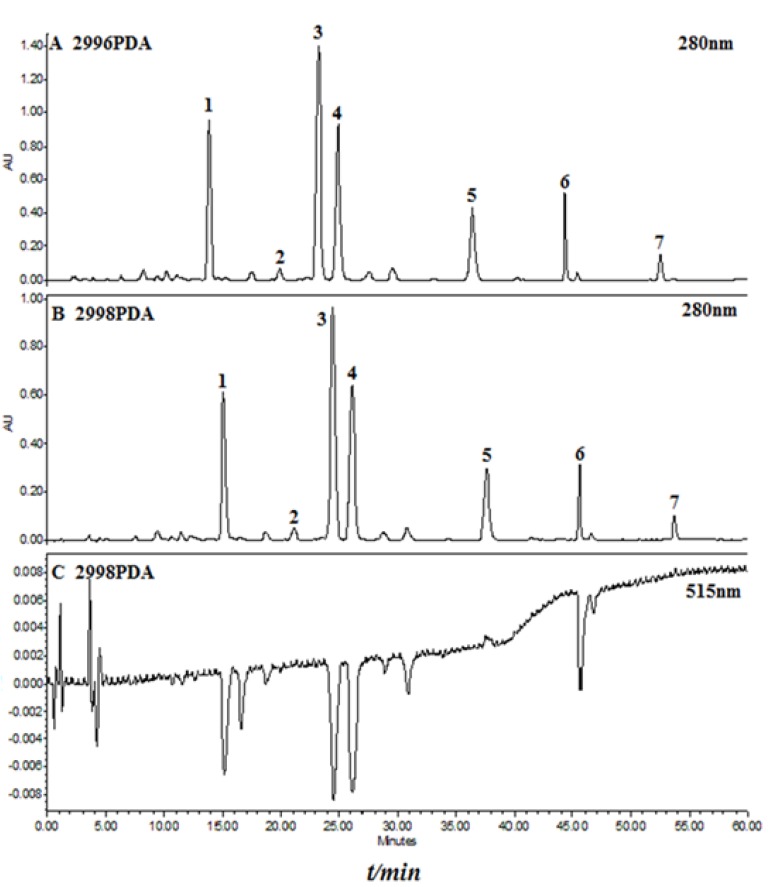
Liquid chromatographic analysis of methanol extract of *O. indicum* (**A**), together with the online post-column DPPH chromatogram (**C**) depicted as negative peaks. Four major active compounds were deduced by comparison of retention times with the chromatogram at 280 nm (**B**): (**1**) baicalein-7-*O*-gentiobioside; (**2**) chrysin-7-*O*-gentiobioside; (**3**) baicalein-7-*O*-glucoside; (**4**) baicalin; (**5**) chrysin-7-*O*-glucuronide; (**6**) baicalein; (**7**) chrysin.

Figure 2 shows the online post-column HPLC-DPPH assay of a methanol extract of Semen Oroxyli. It could be concluded from the chromatograms that nine negative peaks could be found in the DPPH chromatogram (515 nm), including four major flavones. They are baicalein-7-*O*-gentiobioside (**1**), baicalein-7-*O*-glucoside (**3**), baicalin (**4**), and baicalein (**6**) which is consistent with our previous results in an off-line DPPH assay [12]. Three other major flavones which do not contain hydroxyl groups at C-6 were also inactive in this online post-column HPLC-DPPH assay.

### 2.2. DPPH Spiking HPLC Analysis Method

**Figure 3:**
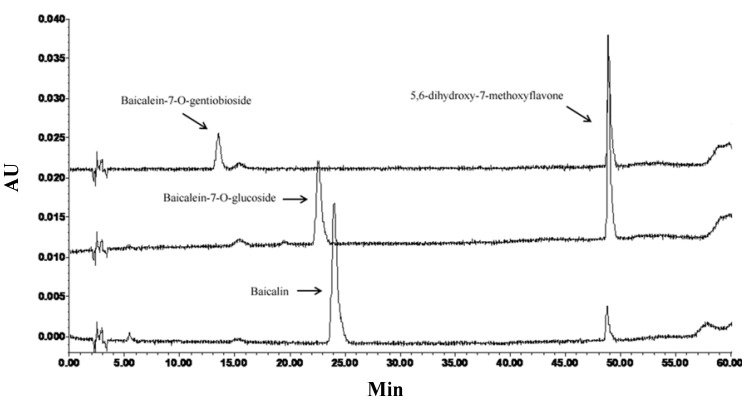
HPLC chromatograms of three flavonoid glycosides after spiking with DPPH.

**Figure 4:**
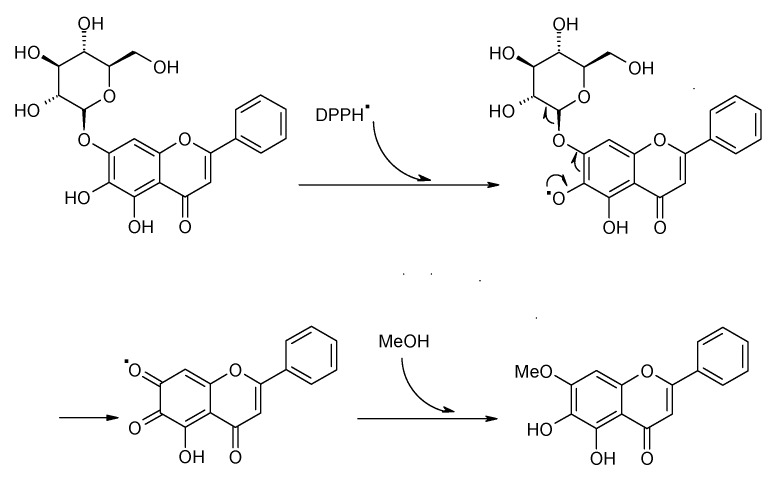
Plausible reaction route of flavonoid glycosides with DPPH, using baicalein-7-*O*-glucoside as an example.

Four active standards were reacted with DPPH, and the chromatograms showed that the peak areas were obviously reduced. In the chromatograms of three flavone glycosides after spiking DPPH, there was a new substance being found at 49 min (Figure 3). After isolation by preparative HPLC, we obtained the newly formed compound. The structure was identified as 5,6-dihydroxy-7-methoxyflavone by ^1^H-NMR, NOE and MS spectra. A plausible reaction mechanism was deduced, as show in Figure 4. It could be concluded from both our previous on-line and off-line antioxidant activity assays [12] that the hydroxyl at C-6 was an essential group for antioxidative activity in the DPPH assay. In this experiment, the reaction mechanism supported this conclusion.

## 3. Experimental

### 3.1. General Information

DPPH was bought from Sigma-Aldrich (St Louis, MO, USA). Methanol (Tjshield, Tianjin, China) and acetonitrile (Fisher, Waltham, MA, USA) used for analytical HPLC were of chromatographic grade. The other chemicals such as methanol for extraction and phosphoric acid were all of analytical purity, which were purchased from Beijing Chemical Works (Beijing, China). All aqueous solutions were prepared with pure water produced by Milli-Q water (18.2 MΩ) system (Millipore, Bedford, MA, USA). Standards of baicalein-7-*O*-gentiobioside, baicalein-7-*O*-glucoside, chrysin-7-*O*-gentiobioside, and chrysin-7-*O*-glucuronide were previously isolated from *O. indicum* in our lab and their structures were characterized by UV, MS, and NMR spectroscopy. The purity of each compound was determined to be more than 98%, according to HPLC analysis based on a peak area normalization method. Baicalin and baicalein were purchased from the National Institutes for Food and Drug Control (Beijing, China). Chrysin was purchased from Sigma-Aldrich. NMR spectra were recorded on a Varian Mercury-400 spectrometer, with TMS as internal standard. ESI-MS was obtained on an Agilent G2451AA 6320 Ion Trap LC/MS system.

### 3.2. Plant Materials

The seeds of *Oroxylum indicum* were collected from Puer City, Yunnan Province of China, in October of 2009, and identified by Prof. Bin Yang, Institute of Chinese Materia Medica, China Academy of Chinese Medical Sciences. A voucher specimen (No. 20090901-PE) has been deposited at the herbarium of the Institute of Chinese Materia Medica, China Academy of Chinese Medical Sciences.

### 3.3. Preparation of Standard and Sample Solutions

The standard solution was prepared by dissolving each standard in methanol at the concentrations of 61.00 μg/mL (baicalein-7-*O*-gentiobioside), 218.00 μg/mL (baicalein-7-*O*-glucoside), 20.70 μg/mL (baicalein), 276.00 μg/mL (baicalin), 28.80 μg/mL (chrysin-7-*O*-gentiobioside), 170.00 μg/mL (chrysin-7-*O*-glucuronide), and 7.50 μg/mL (chrysin).

An accurately weighed powder sample (40 mesh, 500 mg) was suspended in methanol (25 mL), and extracted under ultrasound for 30 min (40 kHZ, 100 W), and then cooled to room temperature. The extracted solution was adjusted to the original weight using methanol, and then filtered through a 0.22 μm filter before injection.

### 3.4. Preparation of DPPH Stock Solution

DPPH (2.23 mg) was dissolved in methanol (500 mL) (1.0 × 10^−5^ mol/L), and it was prepared freshly before daily use. The DPPH stock solution was kept at room temperature in darkness.

### 3.5. HPLC Analysis

Experiments were performed on a Waters Alliance system, consisting of a Waters 2695 pump, a Waters 2996 diode array detector, an auto sampler, a column oven, and an Empower ^TM^ 3 workstation. An Agilent Zorbax Extend-C18 column (5 μm, 4.6 mm × 250 mm, i.d.) was used. Column temperature was controlled at 30 °C. The mobile phase consisted of water–85% phosphoric acid (99.7:0.3, v/v) (solvent A), CH_3_OH (solvent B) and CH_3_CN (solvent C), applied in the following gradient elution: 75%–69% A, 10%–11.5% B and 15%–19.5% C at 0–30 min; 69%–67% A, 11.5%–12% B and 19.5%–21% C at 30–35 min; 67%–54% A, 12%–15% B and 21%–31% C at 35–40 min; and 54%–40% A, 15%–17% B and 31%–43% C at 40–60 min. The flow rate was 1.0 mL/min. Spectra were recorded from 200 to 400 nm while the chromatogram was acquired at 280 nm.

### 3.6. Online Post-Column HPLC-DPPH Assay

**Figure 5:**
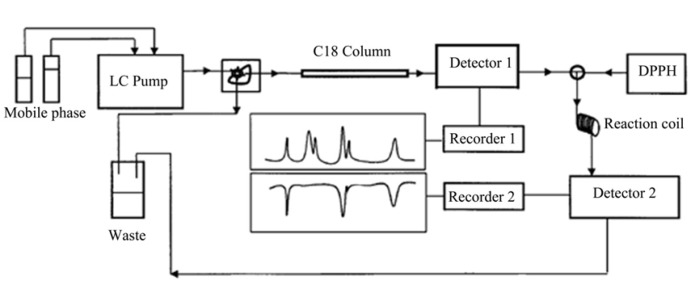
Instrument set-up for on-line post-column HPLC-DPPH assay.

The online post-column HPLC-DPPH assay was carried out according to the reported methods [14,15,16]. Antioxidative activity was evaluated by post-column reaction with DPPH solution supplied by a second HPLC pump (Waters 515, USA) at a flow rate of 0.4 mL/min. The sample-DPPH mixture was passed through a reaction coil (PEEK, 15 m × 0.25 mm) before measurement. The DPPH radical-scavenging detection chromatogram was detected as a negative peak at 515 nm with a Waters 2998 detector, and the chromatogram was recorded on Empower ^TM^ 3 workstation (Waters, USA). Instrumental setup was similar to that of earlier system (Figure 5) [19].

### 3.7. DPPH Spiking HPLC Assay

Two hundred microliters of DPPH stock solution reacted with each 200 μL sample solutions for 60 min, and then the mixture were passed through a 0.22 μm filter and subjected to HPLC analysis. The new compound formed during the assay was identified as 5,6-dihydroxy-7-methoxyflavone. ^1^H-NMR (acetone-*d*_6_, 400 MHz): *δ*_H_ 8.07 (2H, m, H-2', 6'), 7.59 (3H, m, H-3', 4', 5'), 6.79 (1H, br s, H-3), 6.90 (1H, br s, H-8), 3.99 (3H, s, OMe); (+)-ESIMS *m*/*z* 285.0 [M+H]^+^. 

## 4. Conclusions

An online HPLC-DPPH method has been applied for the screening antioxidants from methanol extract of Semen Oroxyli. This screening method seems to be useful for the detection of antioxidants in complex mixtures because of its high sensitivity and simple operation. Four flavones, baicalein-7-*O*-gentiobioside, baicalein-7-*O*-glucoside, baicalein and baicalin, were identified and characterized as active components in Semen Oroxyli. The results are in accord with our previous conventional off-line DPPH assay [12]. The greatest benefit of the online post-column HPLC-DPPH is that, besides the quantification of markers by UV detection, the radical scavenging activity can be measured simultaneously. The activity-integrated quantitative method could be useful for quality control of traditional Chinese medicine, which reflected the real quality.

Despite the fact that abundant data have been reported about the antioxidant activity of flavonoids, the relationship between the structure and activity is still not quite clear [20]. The number and location of hydroxyl groups were considered important factors affecting the antioxidant potency. In the present study, the oxidation product, 5,6-dihydroxy-7-methoxyflavone, was obtained in the DPPH spiking HPLC experiments. Based on the product a plausible mechanism has been proposed (Figure 4). It could be concluded that an additional hydroxyl at C-6 in 5,7-dihydroxyflavones plays a very important role in the DPPH assay results.
