# Correction: Yan, R.Y., *et al.* HPLC-DPPH Screening Method for Evaluation of Antioxidant Compounds Extracted from Semen Oroxyli. *Molecules* 2014, 19, 4409–4417

**DOI:** 10.3390/molecules21010125

**Published:** 2016-01-21

**Authors:** Renyi Yan, Yangyang Cao, Bin Yang

**Affiliations:** 1 Institute of Chinese Materia Medica, China Academy of Chinese Medical Sciences, Beijing 100700, China; yanry2009@163.com (R.Y.); caoyangyanghebei@163.com (Y.C.); 2 State Key Laboratory of Dao-di Herbs, China Academy of Chinese Medical Sciences, Beijing 100700, China

The authors wish to inform readers that there is an error in the chemical structures shown in Figure 4 of this paper [1]. The structure shown in the bottom left corner is an extremely unlikely type of radical. Following the proposed route, it should yield an ortho-quinone (without the unpaired electron) and a pyranosyl sugar free radical. The ortho-quinone should then undergo nucleophilic attack by MeOH to form the product. The corrected Figure 4 is shown below.

**Figure 4:**
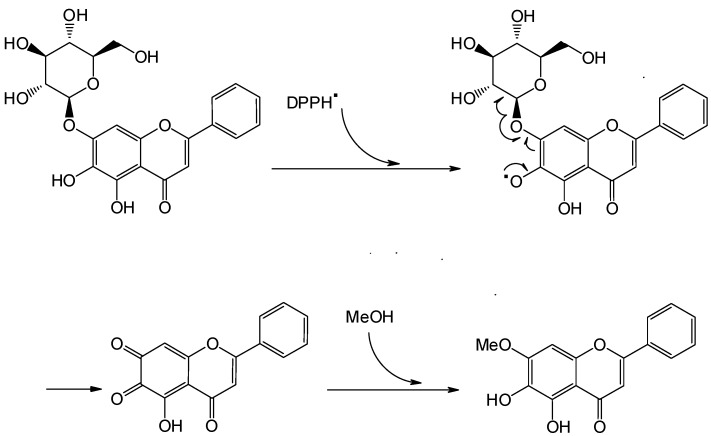
Plausible reaction route of flavonoid glycosides with DPPH, using baicalein-7-*O*-glucoside as an example.

The authors apologize for any inconvenience caused to the readers by these changes. We will update the paper [1] and the original will remain available on the article webpage.
